# An Alternative Method for Generating Arynes from *ortho*-Silylaryl Triflates: Activation by Cesium Carbonate in the Presence of a Crown Ether

**DOI:** 10.3390/molecules200610131

**Published:** 2015-06-01

**Authors:** Suguru Yoshida, Yuki Hazama, Yuto Sumida, Takahisa Yano, Takamitsu Hosoya

**Affiliations:** Laboratory of Chemical Bioscience, Institute of Biomaterials and Bioengineering, Tokyo Medical and Dental University, 2-3-10 Kanda-Surugadai, Chiyoda-ku, Tokyo 101-0062, Japan; E-Mails: s-yoshida.cb@tmd.ac.jp (S.Y.); yuki.h.05261986@gmail.com (Y.H.); yuto.sumida@riken.jp (Y.S.); yano@acls.titech.ac.jp (T.Y.)

**Keywords:** aryne, *ortho*-silylaryl triflate, cesium carbonate, crown ether

## Abstract

An alternative method for generating arynes from *ortho*-silylaryl triflates using cesium carbonate and 18-crown-6 is reported. The method was efficiently applied to a variety of reactions between several arynes and arynophiles. We also demonstrated that the efficiency of aryne generation is significantly affected by the alkali metal countercation of the carbonate.

## 1. Introduction

Arynes are highly reactive intermediates useful for preparing diverse aromatic compounds [1,2,3,4,5,6,7,8]. Novel transformations of arynes have been achieved and applied to the synthesis of a wide range of complex aromatic compounds. In line with the growing importance of aryne chemistry, a variety of methods and precursors to generate arynes have been developed. In particular, activation of *ortho*-silylaryl triflates with a fluoride ion is one of the most widely-used methods for generating arynes [9]. Due to the increased availability of *ortho*-silylaryl triflates [10,11], numerous transformations via arynes have been developed based on this method [12,13,14,15,16,17,18,19,20,21,22,23,24].

As a part of our studies focusing on highly strained alkynes, including arynes [24,25,26,27,28,29,30,31,32,33,34], we have been working on a project to develop new aryne generation methods. For example, we have recently succeeded in efficiently generating arynes from *ortho*-iodoaryl triflates bearing sensitive functional groups using a trimethylsilylmethyl Grignard reagent as an activator [30,31,33] instead of conventional activators such as *n*-butyllithium [35] or a turbo-Grignard reagent [36]. Herein, we report that cesium carbonate, in the presence of a crown ether, serves in place of a fluoride ion as an efficient activator for generating arynes from *ortho*-silylaryl triflates.

## 2. Results and Discussion

We first screened efficient conditions for generating benzyne from 2-(trimethylsilyl)phenyl triflate (**1a**) without the use of a fluoride ion and in the presence of benzyl azide (**2**), which was employed as an arynophile. Consequently, we found that cesium carbonate slowly triggers the generation of benzyne from **1a** to afford benzotriazole **3** (Table 1, entries 1–4). While the reaction in tetrahydrofuran at 25 °C for 24 h resulted in the generation of a trace amount of benzyne with the recovery of a significant amount of **1a** (entry 1), performing the reaction with heating at 60 °C improved the efficiency (entry 2). Further improvement was observed by switching solvent from tetrahydrofuran to acetonitrile (entries 3 and 4). After extensive screening of the conditions to further enhance the efficiency of benzyne generation from **1a** using cesium carbonate, we found that addition of 18-crown-6-ether dramatically accelerates benzyne generation, affording the product **3** in high yield, even when the reaction is performed at 25 °C (entry 5) [37]. Among the various solvents examined, tetrahydrofuran and 1,2-dimethoxyethane gave the best results (entries 5–9). Conversely, generation of benzyne was not observed when cesium carbonate was replaced by cesium bicarbonate (entry 10), which was previously used concomitantly with cesium carbonate and 18-crown-6 to generate benzyne from 2-(trimethylsilyl)phenyl nonaflate with moderate efficiency [38]. Moreover, the efficiency of the reaction was reduced when potassium carbonate and 18-crown-6, which can retain a potassium cation inside the molecule, were used (entry 11). In this case, the yield of **3** was improved by using an increased amount of 18-crown-6 (entry 12). Treatment of **1a** with tripotassium phosphate in the presence of 18-crown-6 also triggered benzyne generation, albeit slowly (entry 13). Although the efficiency of benzyne generation from **1a** mediated by cesium carbonate and 18-crown-6 was slightly inferior to the conventional methods using potassium fluoride and 18-crown-6 (entry 14) or cesium fluoride alone (entry 15), the newly established conditions are worth exploring for optimization of the reactions that use arynes generated from *ortho*-silylaryl triflates.

The optimized reaction conditions were applicable to the reactions between benzyne and various arynophiles (Table 2). Diels–Alder reaction of benzyne generated from **1a** with furan (**4**), 2,5-dimethylfuran (**6**), or *N*-phenylpyrrole (**8**) provided the corresponding cycloadducts **5a**, **7**, and **9**, respectively, in good yields (entries 1–3). Nitrone **10** also reacted with benzyne to afford cycloadduct **11** efficiently (entry 4). Amination of benzyne with morpholine (**12**) proceeded smoothly to yield *N*-phenylmorpholine (**13**) in good yield (entry 5).

**Table 1:** Base-mediated benzyne generation from 2-(trimethylsilyl)phenyl triflate (**1a**).
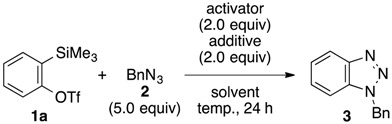

Entry	Activator	Additive	Solvent	Temp. (°C)	Yield (%) ^a^
1	Cs_2_CO_3_	–––	THF	25	1
2	Cs_2_CO_3_	–––	THF	60	32
3	Cs_2_CO_3_	–––	MeCN	25	38
4	Cs_2_CO_3_	–––	MeCN	60	79
5	Cs_2_CO_3_	18-crown-6	THF	25	88 (86) ^b^
6	Cs_2_CO_3_	18-crown-6	MeCN	25	72
7	Cs_2_CO_3_	18-crown-6	CH_2_Cl_2_	25	13
8	Cs_2_CO_3_	18-crown-6	toluene	25	59
9	Cs_2_CO_3_	18-crown-6	DME	25	88
10	CsHCO_3_	18-crown-6	THF	25	0
11	K_2_CO_3_	18-crown-6	THF	25	37
12 ^c^	K_2_CO_3_	18-crown-6	THF	25	76
13	K_3_PO_4_	18-crown-6	THF	25	37
14	KF	18-crown-6	THF	25	99
15	CsF	–––	MeCN	25	quant.

^a^ Yields determined by ^1^H-NMR analyses unless otherwise noted; ^b^ Isolated yield in parentheses; ^c^ 4.0 equivalents of 18-crown-6 were used.

**Table 2:** Reactions of benzyne generated from **1a** with various arynophiles.
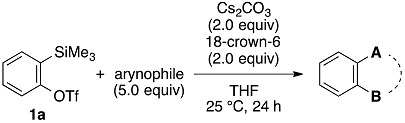

Entry	Arynophile		Product		Yield (%) ^a^
1	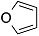	**4**	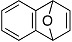	**5a**	73
2	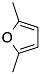	**6**	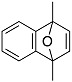	**7**	87
3	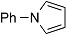	**8**	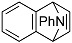	**9**	74
4	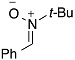	**10**	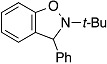	**11**	84
5	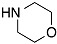	**12**	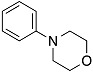	**13**	75

^a^ Isolated yields.

The aryne generation method mediated by cesium carbonate and 18-crown-6 was also successfully applied to generate arynes from various *ortho*-(trimethylsilyl)aryl triflates, and this was demonstrated in the reaction with **4** (Table 3). Indeed, 3- and 4-methoxybenzynes were generated efficiently from the corresponding *ortho*-silylaryl triflates **1b** and **1c**, respectively, to provide the cycloadducts **5b** and **5c** in high yields (entries 1 and 2). Reactions of 3- and 4-methylbenzyne precursors, **1d** and **1e**, as well as 2,3- and 1,2-naphthalyne precursors, **1f** and **1g**, also proceeded smoothly to afford the cycloadducts **5d**–**g** in high yields (entries 3–6).

**Table 3:** Cycloaddition of various arynes with furan.
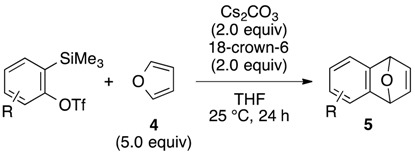

Entry	Arynophile		Product		Yield (%) ^a^
1	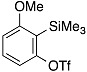	**1b**	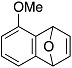	**5b**	89
2	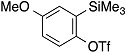	**1c**	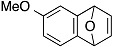	**5c**	86
3	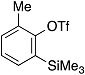	**1d**	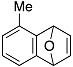	**5d**	88
4	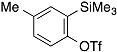	**1e**	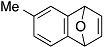	**5e**	89
5	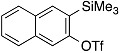	**1f**	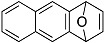	**5f**	76
6	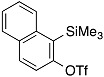	**1g**	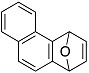	**5g**	85

^a^ Isolated yields.

**Figure 1:**
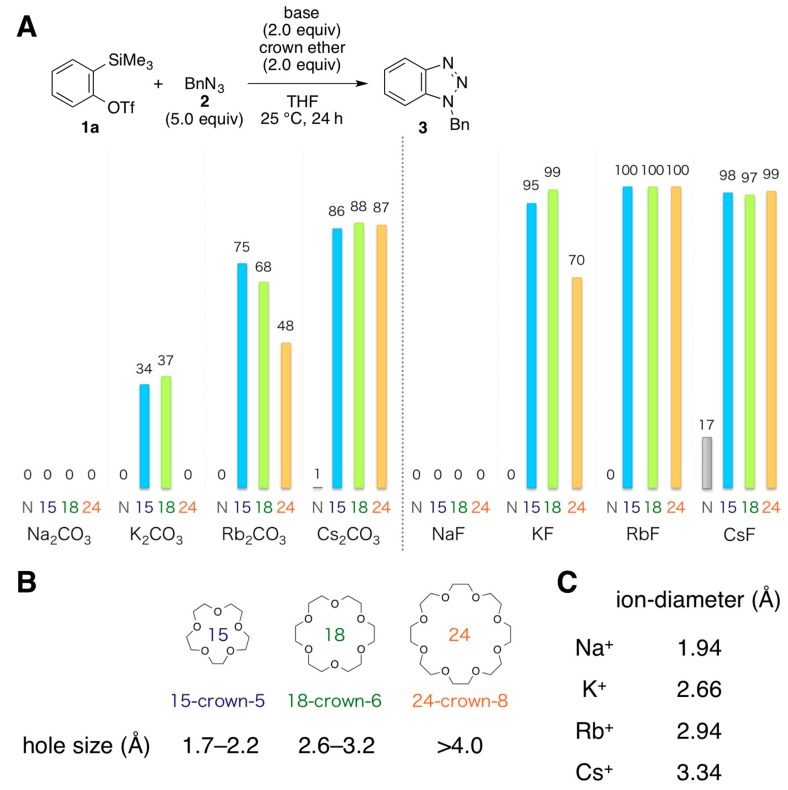
Generation of benzyne from **1a** using various alkali metal carbonates or fluorides and crown ethers. (**A**) Efficiency of the reactions between **1a** and **2** using various bases in combination with different crown ethers (N: without crown ethers; 15: with 15-crown-5; 18: with 18-crown-6; 24: with 24-crown-8). Yields determined by ^1^H-NMR analyses; (**B**) Hole size of crown ethers; (**C**) Diameter of alkali metal ions.

We then turned our attention to the remarkable effect elicited by 18-crown-6-ether, which is able to retain a potassium ion inside the molecule or alternatively coordinate a cesium ion to form a sandwich-type complex [39,40,41,42]. To examine the effects of the countercations of the bases, and the ring size of the crown ether, the efficiencies of benzyne generation from *ortho*-silylphenyl triflate **1a** were compared using several alkali metal carbonates (Na_2_CO_3_, K_2_CO_3_, Rb_2_CO_3_, and Cs_2_CO_3_) or fluorides (NaF, KF, RbF, and CsF) in combination with any one of three crown ethers, 15-crown-5, 18-crown-6, and 24-crown-8, in the presence of benzyl azide (**2**) (Figure 1A). Consequently, the yield of benzotriazole **3**, which reflects the efficiency of benzyne generation, increased as the size of the alkali metal cation of the carbonates became larger. For instance, when 15-crown-5 was employed, the order of the yields of **3** was Na (0%) < K (34%) < Rb (75%) < Cs (86%). A similar trend was observed when 18-crown-6 or 24-crown-8 was used. Fluoride ion-mediated benzyne generation showed the same tendency, Na << K < Rb ≈ Cs, although higher yields of **3** were generally obtained compared with those observed in the carbonate-mediated conditions. These results indicate that the countercation of the base also plays an important role in activating *ortho*-silylaryl triflate for benzyne generation. Moreover, although the use of a crown ether having a hole size smaller than the size of a metal ion (Figure 1B,C) was prone to increase the efficiency of benzyne generation from **1a**, the use of a larger crown ether, such as 24-crown-8, was less effective, particularly when K_2_CO_3_, Rb_2_CO_3_, or KF was used as the base.

**Figure 2:**
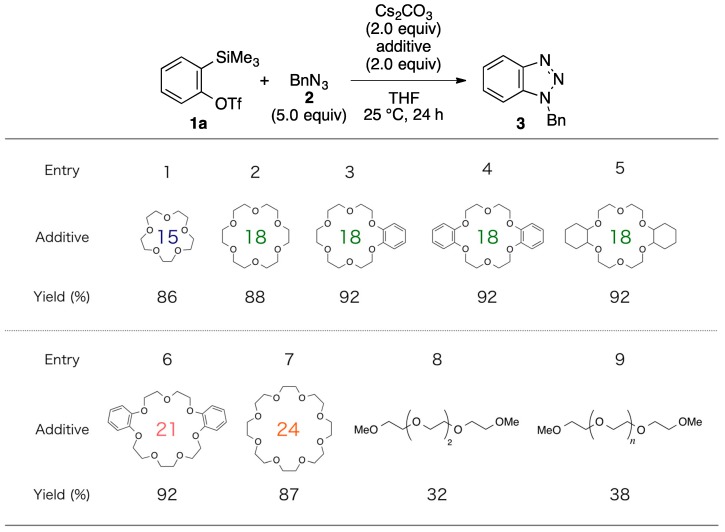
Efficiency of the reactions between **1a** and **2** using cesium carbonate in combination with different ethers. Yields determined by ^1^H-NMR analyses.

Various other crown ethers, regardless of their ring size and benzene- or cyclohexane-linked structure, also effectively supported the cesium carbonate-mediated generation of benzyne from **1a** (Figure 2, entries 1–7). On the other hand, the use of acyclic tetraethylene glycol dimethyl ether or polyethylene glycol dimethyl ether (average molecular weight 240) instead of the crown ether drastically decreased the efficiency (entries 8 and 9). These results suggest that an appropriate complexation between an alkali metal carbonate and a crown ether, such as between cesium carbonate and 18-crown-6, assists the smooth liberation of the triflyloxy anion.

## 3. Experimental Section

### 3.1. General Remarks

All reactions were performed in dried glassware under an argon atmosphere unless otherwise noted. All chemical reagents used were commercial grade and used as received. Aryne precursors **1a**–**1g** were purchased from Tokyo Chemical Industry Co., Ltd. (Tokyo, Japan). Analytical thin-layer chromatography (TLC) was performed on precoated (0.25 mm) silica-gel plates (Silica Gel 60 F_254_, Cat. No. 1.05715, Merck Millipore, Darmstadt, Germany). Column chromatography was conducted using a ZIP sphere cartridge [silica], 10 g (Cat. No. 445-1000-FZ-20, Biotage^®^, Uppsala, Sweden), with a medium pressure liquid chromatograph (W-Prep 2XY A-type Yamazen, Osaka, Japan). ^1^H-NMR spectra were obtained with an AVANCE 400 spectrometer or an AVANCE 500 spectrometer at 400 or 500 MHz, respectively (Bruker BioSpin K.K., Karlsruhe, Germany). ^13^C-NMR spectra were obtained with a Bruker AVANCE 500 spectrometer at 126 MHz. CDCl_3_ (Cat. No. 368651000, Acros Organics, Geel, Belgium) was used as a solvent for obtaining NMR spectra. ^1^H-NMR yields were determined using 1,1,2,2-tetrachloroethane as an internal standard. The spectra obtained for products **3** [28], **5a** [43], **5b**–**d** [44], **5e** [45], **5f** [46], **5g** [47], **7** [28], **9** [28], **11** [28], and **13** [48] were identical to those reported in the corresponding references.

### 3.2. Typical Procedure for Aryne Generation from ortho-Silylaryl Triflate with Cesium Carbonate in the Presence of 18-Crown-6

To a mixture of 2-(trimethylsilyl)phenyl triflate (**1a**, 150 mg, 0.503 mmol) and benzyl azide (**2**, 333 mg, 2.50 mmol) dissolved in tetrahydrofuran (2.5 mL) was added cesium carbonate (326 mg, 1.00 mmol) and 18-crown-6-ether (265 mg, 1.00 mmol) at room temperature. After stirring for 24 h at 25 °C, water (10 mL) was added to the mixture. The mixture was then extracted with ethyl acetate (15 mL × 3), and the combined organic extract was washed with brine (10 mL), dried (Na_2_SO_4_), and, after filtration, the filtrate was concentrated under reduced pressure. The residue was purified by column chromatography (silica-gel 10 g, *n*-hexane/EtOAc = 5/1) to give 1-benzyl-1*H*-benzo[*d*][1,2,3]triazole (**3**, 90.0 mg, 0.431 mmol, 85.6%) as a colorless solid.

## 4. Conclusions

We have demonstrated that cesium carbonate, in the presence of 18-crown-6, triggers the efficient generation of arynes from *ortho*-silylaryl triflates under mild conditions. The method was applicable to a variety of reactions between several arynes and arynophiles. Various crown ethers other than 18-crown-6 were found to be similarly effective, but the efficiency of aryne generation significantly depended upon the alkali metal countercation of the carbonate. Further studies to demonstrate the advantage of this newly developed method are underway.

## References

[1] Hoffmann R.W. (1967). Dehydrobenzene and Cycloalkynes.

[2] Pellissier H., Santelli M. (2003). The use of arynes in organic synthesis. Tetrahedron.

[3] Yoshida H., Takaki K. (2012). Multicomponent Coupling Reaction of Arynes for Construction of Heterocyclic Skeletons. Heterocycles.

[4] Yoshida H., Takaki K. (2012). Aryne Insertion Reactions into Carbon-Carbon σ-Bonds. Synlett.

[5] Gampe C.M., Carreira E.M. (2012). Arynes and Cyclohexyne in Natural Product Synthesis. Angew. Chem. Int. Ed..

[6] Tadross P.M., Stoltz B.M. (2012). A Comprehensive History of Arynes in Natural Product Total Synthesis. Chem. Rev..

[7] Dubrovskiy A.V., Markina N.A., Larock R.C. (2013). Use of benzynes for the synthesis of heterocycles. Org. Biomol. Chem..

[8] Hoffmann R.W., Suzuki K. (2013). A “Hot, Energized” Benzyne. Angew. Chem. Int. Ed..

[9] Himeshima Y., Sonoda T., Kobayashi H. (1983). Fluoride-Induced 1,2-Elimination of *O*-Trimethylsilylphenyl Triflate to Benzyne under Mild Conditions. Chem. Lett..

[10] Bronner S.M., Garg N.K. (2009). Efficient Synthesis of 2-(Trimethylsilyl)phenyl Trifluoromethanesulfonate: A Versatile Precursor to *o*-Benzyne. J. Org. Chem..

[11] Peña D., Cobas A., Pérez D., Guitián E. (2002). An Efficient Procedure for the Synthesis of *ortho*-Trialkylsilylaryl Triflates: Easy Access to Precursors of Functionalized Arynes. Synthesis.

[12] Candito D.A., Lautens M. (2011). Stereoselective Nickel-Catalyzed [2+2+2] Cycloaddition of Enynes and Arynes. Synlett.

[13] Smith A.B., Kim W.-S. (2011). Diversity-oriented synthesis leads to an effective class of bifunctional linchpins uniting anion relay chemistry (ARC) with benzyne reactivity. Proc. Natl. Acad. Sci. USA.

[14] Allan K.M., Gilmore C.D., Stoltz B.M. (2011). Benzannulated Bicycles by Three-Component Aryne Reactions. Angew. Chem. Int. Ed..

[15] Ikawa T., Nishiyama T., Shigeta T., Mohri S., Morita S., Takayanagi S., Terauchi Y., Morikawa Y., Takagi A., Ishikawa Y. (2011). *ortho*-Selective Nucleophilic Addition of Primary Amines to Silylbenzynes: Synthesis of 2-Silylanilines. Angew. Chem. Int. Ed..

[16] Yoshioka E., Kohtani S., Miyabe H. (2011). A Multicomponent Coupling Reaction Induced by Insertion of Arynes into the C=O Bond of Formamide. Angew. Chem. Int. Ed..

[17] Yoshida H., Yoshida R., Takaki K. (2013). Synchronous Ar–F and Ar–Sn Bond Formation through Fluorostannylation of Arynes. Angew. Chem. Int. Ed..

[18] Saito N., Nakamura K., Shibano S., Ide S., Minami M., Sato Y. (2013). Addition of Cyclic Ureas and 1-Methyl-2-oxazolidone to Pyridynes: A New Approach to Pyridodiazepines, Pyridodiazocines, and Pyridooxazepines. Org. Lett..

[19] Goetz A.E., Garg N.K. (2013). Regioselective reactions of 3,4-pyridynes enabled by the aryne distortion model. Nat. Chem..

[20] Hall C., Henderson J.L., Ernouf G., Greaney M.F. (2013). Tandem thia-Fries rearrangement—Cyclisation of 2-(trimethylsilyl)phenyl trifluoromethanesulfonate benzyne precursors. Chem. Commun..

[21] Bhojgude S.S., Thangaraj M., Suresh E., Biju A.T. (2014). Tandem [4 + 2]/[2 + 2] Cycloaddition Reactions Involving Indene or Benzofurans and Arynes. Org. Lett..

[22] Liu F.-L., Chen J.-R., Zou Y.-Q., Wei Q., Xiao W.-J. (2014). Three-Component Coupling Reaction Triggered by Insertion of Arynes into the S=O Bond of DMSO. Org. Lett..

[23] Pandya V.G., Mhaske S.B. (2014). Transition-Metal-Free C–S Bond Formation: A Facile Access to Aryl Sulfones from Sodium Sulfinates via Arynes. Org. Lett..

[24] Yoshida S., Hosoya T. (2013). Synthesis of Diverse Aromatic Oxophosphorus Compounds by the Michaelis–Arbuzov-type Reaction of Arynes. Chem. Lett..

[25] Kii I., Shiraishi A., Hiramatsu T., Matsushita T., Uekusa H., Yoshida S., Yamamoto M., Kudo A., Hagiwara M., Hosoya T. (2010). Strain-promoted double-click reaction for chemical modification of azido-biomolecules. Org. Biomol. Chem..

[26] Yoshida S., Shiraishi A., Kanno K., Matsushita T., Johmoto K., Uekusa H., Hosoya T. (2011). Enhanced clickability of doubly sterically-hindered aryl azides. Sci. Rep..

[27] Yoshida S., Hatakeyama Y., Johmoto K., Uekusa H., Hosoya T. (2014). Transient Protection of Strained Alkynes from Click Reaction via Complexation with Copper. J. Am. Chem. Soc..

[28] Sumida Y., Kato T., Hosoya T. (2013). Generation of Arynes via Ate Complexes of Arylboronic Esters with an *ortho*-Leaving Group. Org. Lett..

[29] Yoshida S., Uchida K., Hosoya T. (2014). Generation of Arynes Triggered by Sulfoxide–Metal Exchange Reaction of *ortho*-Sulfinylaryl Triflates. Chem. Lett..

[30] Yoshida S., Nonaka T., Morita T., Hosoya T. (2014). Modular synthesis of bis- and tris-1,2,3-triazoles by permutable sequential azide-aryne and azide-alkyne cycloadditions. Org. Biomol. Chem..

[31] Yoshida S., Uchida K., Igawa K., Tomooka K., Hosoya T. (2014). An efficient generation method and remarkable reactivities of 3-triflyloxybenzyne. Chem. Commun..

[32] Sumida Y., Harada R., Kato-Sumida T., Johmoto K., Uekusa H., Hosoya T. (2014). Boron-Selective Biaryl Coupling Approach to Versatile Dibenzoxaborins and Application to Concise Synthesis of Defucogilvocarcin M. Org. Lett..

[33] Yoshida S., Uchida K., Hosoya T. (2015). Generation of Arynes Using Trimethylsilylmethyl Grignard Reagent for Activation of *ortho*-Iodoaryl or *ortho*-Sulfinylaryl Triflates. Chem. Lett..

[34] Yoshida S., Karaki F., Uchida K., Hosoya T. (2015). Generation of cycloheptynes and cyclooctynes via a sulfoxide–magnesium exchange reaction of readily synthesized 2-sulfinylcycloalkenyl triflates. Chem. Commun..

[35] Matsumoto T., Hosoya T., Katsuki M., Suzuki K. (1991). New Efficient Protocol for Aryne Generation. Selective Synthesis of Differentially Protected 1,4,5-Naphthalenetriols. Tetrahedron Lett..

[36] Ikawa T., Takagi A., Kurita Y., Saito K., Azechi K., Egi M., Kakiguchi K., Kita Y., Akai S. (2010). Preparation and Regioselective Diels–Alder Reactions of Borylbenzynes: Synthesis of Functionalized Arylboronates. Angew. Chem. Int. Ed..

[B37-molecules-20-10131] 37.Cycloadduct **3** was observed as a sole product and starting material **1a** was completely consumed. In the case of using 0.1 or 1.0 equiv of 18-crown-6, cycloadduct **3** was obtained in 31% or 74% yield, respectively.

[38] Ikawa T., Nishiyama T., Nosaki T., Takagi A., Akai S. (2011). A Domino Process for Benzyne Preparation: Dual Activation of *o*-(Trimethylsilyl)phenols by Nonafluorobutanesulfonyl Fluoride. Org. Lett..

[39] Steed J.W., Atwood J.L. (2000). Supramolecular Chemistry.

[40] Gokel G.W. (1997). Advances in Supramolecular Chemistry.

[41] Pedersen C.J. (1970). Crystalline Salt Complexes of Macrocyclic Polyethers. J. Am. Chem. Soc..

[42] Frensdorff H.K. (1971). Stability Constants of Cyclic Polyether Complexes with Univalent Cations. J. Am. Chem. Soc..

[43] Nishimura T., Kawamoto T., Sasaki K., Tsurumaki E., Hayashi T. (2007). Rhodium-Catalyzed Asymmetric Cyclodimerization of Oxa- and Azabicyclic Alkenes. J. Am. Chem. Soc..

[44] Lautens M., Schmid G.A., Chau A. (2002). Remote Electronic Effects in the Rhodium-Catalyzed Nucleophilic Ring Opening of Oxabenzonorbornadienes. J. Org. Chem..

[45] Fillion E., Trépanier V.É., Heikkinen J.J., Remorova A.A., Carson R.J., Goll J.M., Seed A. (2009). Palladium-Catalyzed Intramolecular Reactions of (*E*)-2,2-Disubstituted 1-Alkenyldimethylalanes with Aryl Triflates. Organometallics.

[46] Chen Y.-L., Sun J.-Q., Wei X., Wong W.-Y., Lee A.W.M. (2004). Generation of Synthetic Equivalents of Benzdiynes from Benzobisoxadisilole. J. Org. Chem..

[47] Best W.M., Collins P.A., McCulloch R.K., Wege D. (1982). Deoxygenation of 1,4-Epoxy-1,4-dihydroarenes with Enneacarbonyldiiron. Aust. J. Chem..

[48] Swapna K., Kumar A.V., Reddy V.P., Rao K.R. (2009). Recyclable Heterogeneous Iron Catalyst for C−N Cross-Coupling under Ligand-Free Conditions. J. Org. Chem..

